# A Case of Myoepithelioma-Like Tumor of the Vulvar Region after Removal of an Inguinal Tumor

**DOI:** 10.70352/scrj.cr.25-0237

**Published:** 2025-08-05

**Authors:** Kei Urakami, Hiroaki Saito, Akimitsu Tanio, Yoichiro Tada, Yoshinori Yamada, Yutaka Yamashiro, Yumi Yamaguchi

**Affiliations:** 1 Division of Gastrointestinal and Pediatric Surgery, Department of Surgery, School of Medicine, Faculty of Medicine, Tottori University, Yonago, Tottori, Japan; 2 Department of Surgery, Japanese Red Cross Tottori Hospital, Tottori, Tottori, Japan

**Keywords:** myoepithelioma-like tumor of the vulvar region, myoepithelioma, inguinal tumor

## Abstract

**INTRODUCTION:**

Myoepithelioma-like tumor of the vulvar region (MELTVR) is a mesenchymal neoplasm first reported in 2015 and typically develops from the inguinal to the vulvar regions of adult women.

**CASE PRESENTATION:**

Here we report the case of a 42-year-old woman who presented with right inguinal tumor. The tumor had recently increased in size continuously. Computed tomography (CT) showed a homogeneous neoplastic lesion along the uterine cord in the right inguinal region and marginal resection was performed. Pathological examination revealed a well-defined tumor. And there were areas of epithelial-like tumor cells arranged in a reticular or cord-like pattern against a background of mucinous stroma, and areas of spindle-shaped cells growing in mucinous substrate with transition from epithelial cells. The nucleus was irregular in size and shape. Necrotic nests were scattered in the tumor. Immunohistological examination showed that the tumor cells were positive for epithelial membrane antigen (EMA), estrogen receptor (ER), and progesterone receptor (PgR). Alpha-smooth muscle actin (α-SMA) was slightly positive. The tumor was negative for cytokeratin AE1/AE3, p63, desmin, CD34, S100, glial fibrillary acidic protein (GFAP), and SOX10. Loss of INI1 protein expression was also confirmed. The patient was suspected of having high-grade myoepithelioma on pathological diagnosis at our hospital. However, immunohistological findings led to the diagnosis of MELTVR. The patient underwent additional wide excision and has been alive 10 months postoperatively without recurrence.

**CONCLUSIONS:**

Due to its rarity, it is difficult to make preoperative diagnosis of MELTVR. Awareness of this condition can contribute to accurate diagnosis and appropriate management in adult female patients presenting with swelling extending from the inguinal to the vulvar regions.

## Abbreviations

EMA
: epithelial membrane antigen
EMC
: extraskeletal myxoid chondrosarcoma
ER
: estrogen receptor
GFAP
: glial fibrillary acidic protein
MELTVR
: myoepithelioma-like tumor of the vulvar region
PgR
: progesterone receptor
α-SMA
: alpha-smooth muscle actin

## INTRODUCTION

MELTVR is a rare soft tissue tumor first reported by Yoshida et al. in 2015.^1)^ It typically arises in the groin or vulvar region of adult women. There are some common diseases showing a bulge in the vulvar region such as Nuck’s cyst, inguinal hernia, endometriosis, and neoplasms including fibrous tumor, angiomyxoma, angiomyofibroblastoma, lipoma, hemangioma, and schwannoma. Due to its rarity, this disease is not well known. Awareness of this condition can contribute to accurate diagnosis and appropriate management in adult female patients presenting with swelling extending from the inguinal to the vulvar regions. In the present report, we presented a case of MELTVR that was diagnosed on postoperative pathological examination. We compare this case with previously reported cases and discuss the prognosis and treatment strategy of MELTVR.

## CASE PRESENTATION

**Fig. 1:**
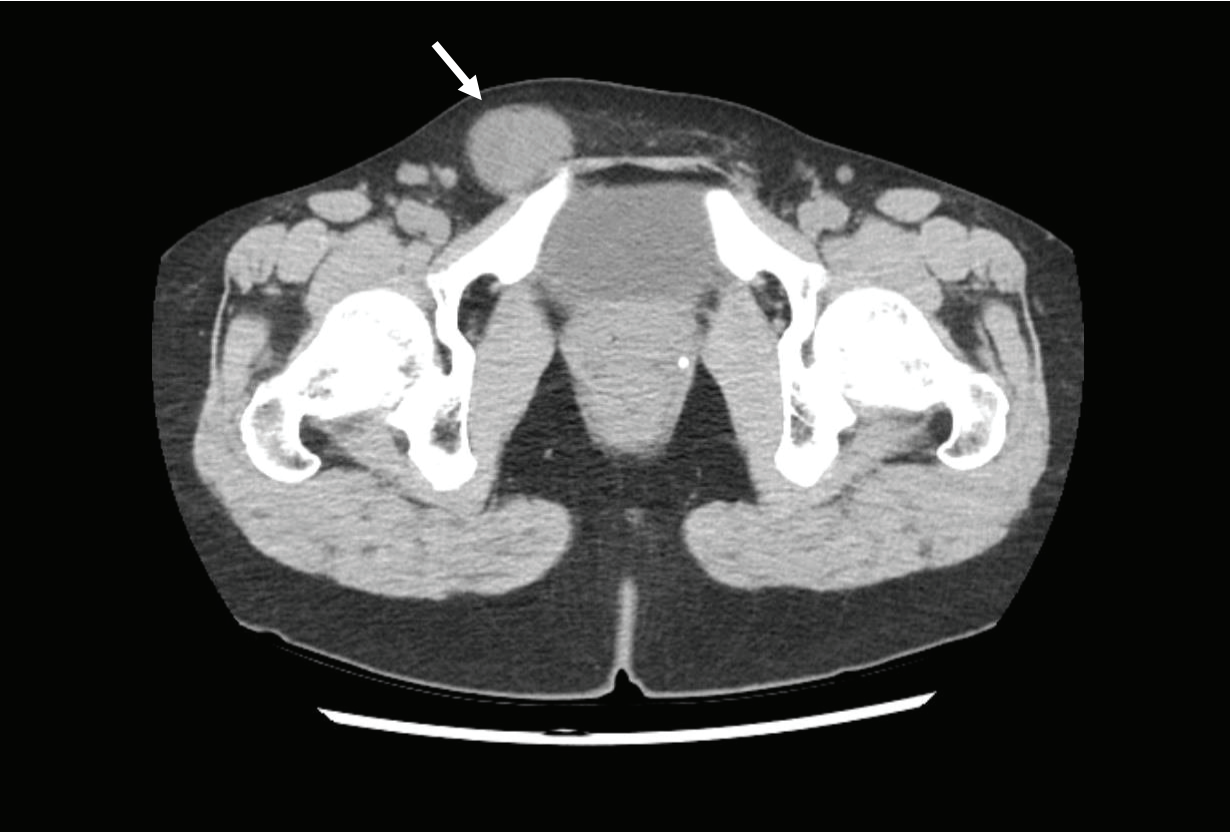
Preoperative CT findings. CT image showing a homogeneous neoplastic lesion measuring 30 × 40 mm along the uterine cord in the right inguinal region.

**Fig. 2:**
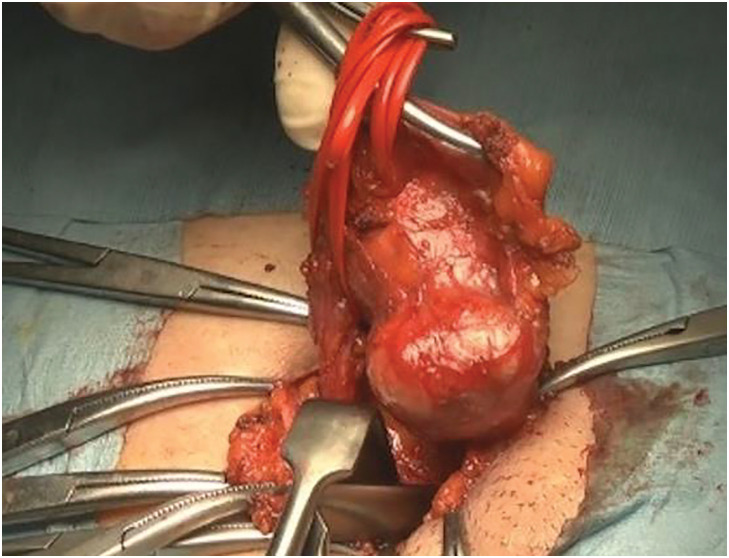
Intraoperative findings. Surgical findings showed that there was a tumor along the uterine cord.

A 42-year-old woman was referred to our hospital because of right inguinal distension that she had been aware of for 1 year. There was no significant past medical, family, or psychosocial history. On physical examination, there was a 30-mm-sized mass in the right inguinal region. It was elastic hard, mobile with mild tenderness, and irreducible. Blood chemical examination was within normal ranges. CT revealed a homogeneous neoplastic lesion along the uterine cord in the right inguinal region (**Fig. 1**). Based on the findings of CT and physical examination, we suspected hydrocele of the canal of Nuck and performed an operation. In this case, MRI and ultrasound examinations were not performed. Laparoscopy revealed that there was no inguinal hernia. The tumor was found along the uterine cord (**Fig. 2**) and had extended to the vulva. We performed marginal resection and Marcy’s method to prevent hernia. The postoperative course was uneventful.

**Fig. 3:**
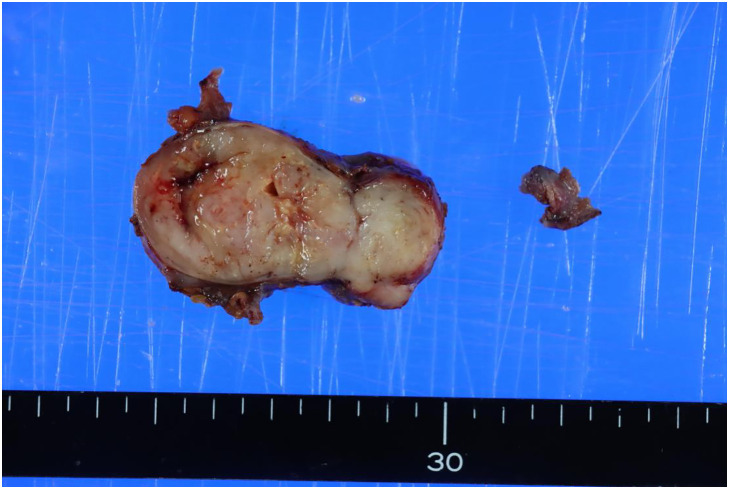
Photograph of the gross specimens. The tumor measuring 30 × 40 × 60 mm with the cut surface having the appearance of grayish white substantial components.

**Fig. 4:**
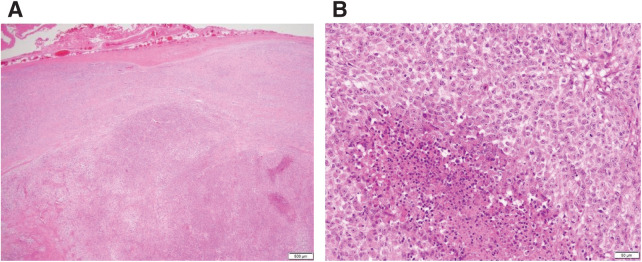
Histopathological findings of the present case. Histopathological findings show that the tumor was well-defined with a fibrous capsule (**A**). There were areas of epithelial-like tumor cells arranged in a reticular or cord-like pattern against a background of mucinous stroma, and areas of spindle-shaped cells growing in a mucinous substrate with transition from epithelial cells (**B**).

**Fig. 5:**
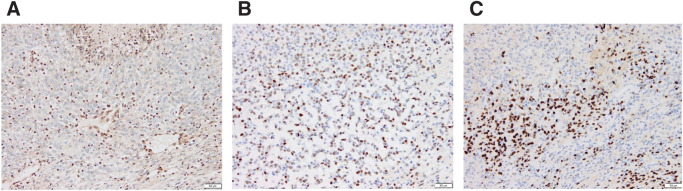
Immunohistochemistry findings of the present case. Photomicrographs of immunohistochemically stained tissue sections. (**A**) Loss of INI1 protein expression was confirmed in tumor cells and tumors were positive for ER (**B**) and PgR (**C**). ER, estrogen receptor; PgR, progesterone receptor

**Fig. 6:**
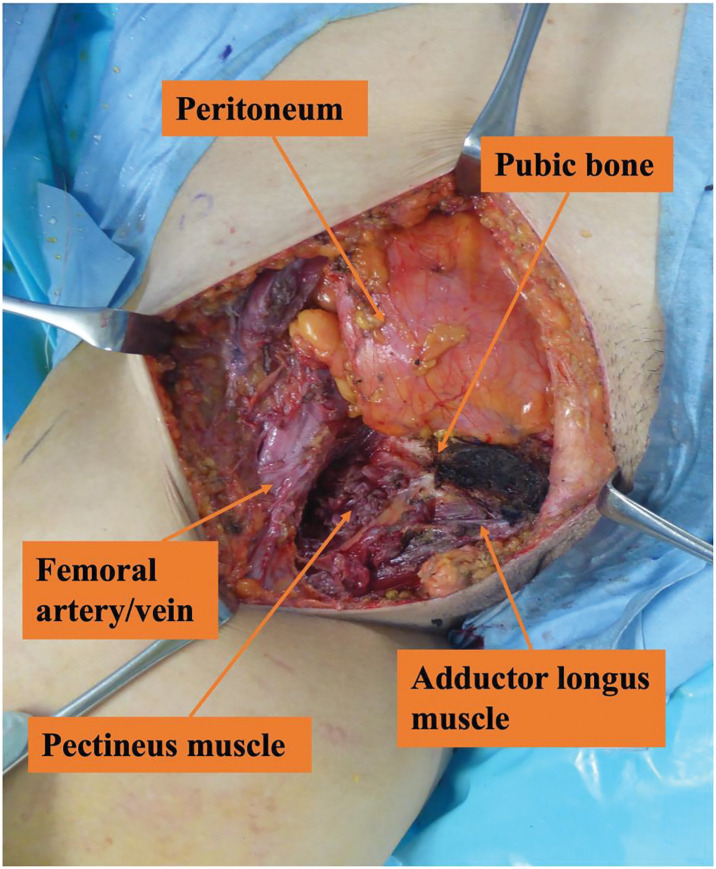
Photograph of extended excision. Surgical findings showed that this is after specimen removal during extended resection.

Macroscopically, the resected specimen measured 30 × 40 × 60 mm with the cut surface having the appearance of grayish white substantial components (**Fig. 3**). Pathological examination revealed a well-defined tumor with a fibrous capsule (**Fig. 4A**). Epithelial-like tumor cells with eosinophilic cytoplasm formed dense growth nests. There were areas of epithelial-like tumor cells arranged in a reticular or cord-like pattern against a background of mucinous stroma, and areas of spindle-shaped cells growing in mucinous substrate with transition from epithelial cells (**Fig. 4B**). The nucleus was irregular in size and shape. Large and small necrotic nests and hemorrhagic cystic degeneration nests were scattered in the tumor. Immunohistological examination showed that the tumor cells were positive for EMA and negative for cytokeratin AE1/AE3, p63, desmin, CD34, S100, GFAP, and SOX10. Loss of INI1 protein expression was also confirmed (**Fig. 5A**). α-SMA was slightly positive. ER and PgR were also positive (**Fig. 5B** and **5C**). Although the pathological findings were consistent with a myoepithelioma, the immunohistochemical findings were not. Based on the pathological features, she was diagnosed with a myoepithelioma variant and was referred to the university hospital for further evaluation. Despite further evaluation at the university hospital, a definitive diagnosis could not be made and there was suspicion of malignant myoepithelioma. Additional resection was planned to ensure an adequate surgical margin, considering the possibility of malignant myoepithelioma. Additional wide excision and reconstruction with anterolateral thigh flap were performed. The resection area was designed as an elongated oval measuring 8 cm vertically and 11 cm horizontally, centered on the tumor site. The resection margin extended medially to the medial border of the rectus abdominis muscle and caudally to a point 2 cm below the inferior edge of the pubic symphysis. The procedure was initiated with a skin incision made slightly to the left of the midline, followed by division of the rectus abdominis muscle to expose the peritoneum. Dissection was then carried out between the rectus abdominis muscle and the peritoneum, proceeding from medial to lateral. On the lateral side, the external oblique, internal oblique, and transversus abdominis muscles were sequentially incised along the skin incision line to expose the peritoneum. Dissection continued along the peritoneal surface and was connected with the medial dissection plane. The dissection was extended caudally, with merger resection of the inguinal ligament. The femoral artery and vein were preserved. Partial resection of the pectineus and adductor longus muscles was performed. Additionally, at the insertion site of the right rectus abdominis on the pubic bone, 2 mm of cortical bone from the superior surface of the pubis was removed by using osteotomes, and the specimen was excised en bloc (**Fig. 6**). There was no residual tumor cell in the resected specimen. The tumor samples resected in our hospital were sent to the National Cancer Center Japan Institute for Cancer Control for further pathological evaluation and diagnosed as MELTVR. The patient remained relapse-free after the lesion was widely excised during 10 months’ follow-up.

## DISCUSSION

MELTVR is a recently described mesenchymal neoplasm which typically arises in the vulvar region of adult women. The clinical manifestation of MELTVR is not specific. Most patients presented with a painless mass or had occasional pain. There are a wide variety of diseases showing a bulge in the vulvar region such as Nuck’s cyst, inguinal hernia, endometriosis, and neoplasms, including fibrous tumor, angiomyxoma, angiomyofibroblastoma, lipoma, hemangioma, and schwannoma^1)^. In the present case, Nuck’s cyst was suspected preoperatively. It has been reported that Nuck’s cyst is associated with endometriosis.^2)^ Additionally, there have been reports of coexisting malignant tumors.^3)^ In this case, resection was performed considering the possibility of endometriosis coexisting with Nuck’s cyst. Although inguinal tumors are rare and were not initially suspected, additional preoperative imaging, such as ultrasound or MRI, might have been warranted. To the best of our knowledge, only 13 MELTVR has been reported so far. Due to its rarity, it is difficult to make accurate preoperative diagnosis. In fact, the accurate preoperative diagnosis of MELTVR was not made in 13 MELTVR patients reported thus far.

The diagnosis of MELTVR can be made by the combination of histological and immunohistochemical features. MELTVR resembles myoepithelioma in histopathologic findings and may sometimes be considered a variant of myoepithelioma, although it is considered an independent tumor.^1)^ Our case was also pathologically considered a myoepithelioma variant in our hospital. MELTVR is pathologically characterized by the presence of intra-tumoral tissue diversity and mucoid stroma, and immunohistochemical findings show the expression of EMA and ER.^1)^ Immunohistochemical characteristics of the 12 reported cases^1,4–6)^ and the present one are summarized in **Table 1**. Although there are no specific immunohistochemical markers, ER and EMA are consistently positive in MELTVR (**Table 1**). **Table 1** shows that PgR-positive may also aid in diagnosis. Meanwhile, myoepithelioma is pathologically characterized by spindle, round, and epithelioid cells growing in a solid, reticular, trabecular, or alveolar pattern in mucoid stroma.^7)^ Immunohistochemical studies are essential for the diagnosis of myoepithelioma, because diverse forms of cell and structure are like those of MELTVR. Myoepithelioma expresses cytokeratin and is positive for myoepithelial markers such as GFAP and S100,^8)^ but these were negative in this case. Furthermore, SOX10 is frequently positive in myoepithelioma,^9)^ but was also negative in this case. Moreover, EMC is another differential disease. EMC histopathologically shows oval to spindle cells in mucous-like matrix,^10)^ but it is relatively uniform and does not have the variety of MELTVR. Therefore, based on pathological and immunohistochemical findings, our case was consistent with MELTVR.

**Table 1:** Immunohistochemical results in 13 MELTVR patients reported thus far

Author (References)	Immunohistochemistry findings									
	PgR	ER	EMA	GFAP	S100	CD34	INI1	AE1/AE3	SMA	Desmin
Yoshida et al.^1)^ (n = 9)	+	+	+	–	–	–	–	+	+	NA
Kaku et al.^4)^ (n = 1)	+	+	+	–	–	–	–	+	–	NA
Kojima et al.^5)^ (n = 1)	NA	+	+	–	–	–	–	–	+	–
Xu et al.^6)^ (n = 1)	NA	+	+	–	–	–	–	–	–	–
Our case (n = 1)	+	+	+	–	–	–	–	–	+	–

EMA, epithelial membrane antigen; ER, estrogen receptor; GFAP, glial fibrillary acidic protein; MELTVR, myoepithelioma-like tumor of the vulvar region; NA, not available; PgR, progesterone receptor

MELTVR is a new disease concept and is likely to have been treated as myoepithelioma thus far. Because a limited number of MELTVR have been reported thus far, optimal treatment remains unclear. Among 12 patients who were diagnosed as MELTVR, all patients underwent surgery (**Table 2**). The initial resection of the 12 reported cases includes 4 cases of marginal resection, 3 cases of intralesional resection, 4 cases of enucleation, and 1 case of wide excision. Two cases of intralesional resection show tumor regrowth. One case of enucleation shows local recurrence 12 years after surgery; 7 of the 12 cases underwent additional extensive resection with or without recurrence, and 1 case underwent marginal resection after recurrence. All patients are recurrence-free and have no apparent local recurrence even after marginal resection. Although there is a possibility that marginal resection may be acceptable as treatment strategy, there are reports of vascular invasion^1)^ or the desirability of adequate margins in cases of local invasion.^11)^ Therefore, wide excision with the purpose of complete resection might be an appropriate treatment strategy. It has been reported that, in cases of malignant soft tissue tumors, performing additional resection within 12 weeks of the initial surgery can help minimize deterioration of prognosis.^12)^ In the present case, because malignancy was suspected, additional resection was performed before the definitive diagnosis of MELTVR was established. Among previously reported cases,^1,4–6)^ none underwent surgery after a confirmed diagnosis of MELTVR. The difficulty in diagnosis and the resulting delay in establishing a definitive diagnosis may have also contributed to the clinical course. Although the malignancy of MELTVR cannot be determined due to the small number of cases, it is assumed that MELTVR has a slow clinical course even in the presence of nuclear atypia, necrosis, or some degree of proliferative capacity. Due to the slow-growing nature of MELTVR, wide resection may be feasible even after a definitive diagnosis is confirmed. Further investigation is required to clearly determine the malignant potential of this disease and adequate operation for this disease.

**Table 2:** Summary of clinical findings of previously reported and present cases of MELTVR

No (References)	Age	Site	Size (mm)	Initial resection	Initial outcome	Additional resection	AC	Metastasis	Final outcome
1 Yoshida et al.^1)^	49	Labium majus	43	Marginal resection	–	–	–	–	NED
2 Yoshida et al.^1)^	42	Labium majus	70	Intralesional resection	Regrowth	Wide excision	–	–	NED
3 Yoshida et al.^1)^	24	Labium majus	33	Enucleation	–	Wide excision	–	–	NED
4 Yoshida et al.^1)^	41	Labium majus	20	Enucleation	Regrowth	Wide excision	–	–	NED
5 Yoshida et al.^1)^	52	Mons pubis	77	Marginal resection	–	Wide excision	–	–	NED
6 Yoshida et al.^1)^	28	Labium majus	30	Enucleation	–	Wide excision	–	–	NED
7 Yoshida et al.^1)^	35	Groin	24	Intralesional resection	–	Wide excision	–	–	NED
8 Yoshida et al.^1)^	65	Groin	32	Marginal resection	–	–	–	–	NED
9 Yoshida et al.^1)^	35	Mons pubis	NA	Intralesional resection	Local recurrence	Marginal resection	–	–	NED
10 Kaku et al.^4)^	31	Mons pubis	20	Enucleation	–	Wide excision	–	–	NED
11 Kojima et al.^5)^	70	Groin	36	Marginal resection	–	–	–	–	NED
12 Xu et al.^6)^	65	Perianal region	55	Wide excision	–	–	–	–	NED
13 Present	42	Groin	40	Marginal resection	–	Wide excision	–	–	NED

AC, adjuvant chemotherapy; MELTVR, myoepithelioma-like tumor of the vulvar region; NED, no evidence of disease

## CONCLUSIONS

We have reported a rare case of MELTVR. Due to its rarity, it is difficult to make a preoperative diagnosis of MELTVR. Awareness of this condition can contribute to accurate diagnosis and appropriate management in adult female patients presenting with swelling extending from the inguinal to the vulvar regions. Further investigation is required to clearly determine the pathological, immunohistochemical and molecular features; malignant potential; and management of this disease.

## ACKNOWLEDGMENTS

We would like to thank Editage (www.editage.jp) for English language editing.

## DECLARATIONS

### Funding

No specific funding was received for this study.

### Authors’ contributions

KU drafted the manuscript. HS critically reviewed the manuscript.

KU and YYamad performed 1st operations.

All authors read and approved the final manuscript and agreed to take responsibility for all aspects of the study.

### Availability of data and materials

The data and materials used in this case report can be made available upon reasonable request to the corresponding author, within the scope of the patient’s consent.

### Ethics approval and consent to participate

This work does not require ethical considerations or approval. Informed consent to participate in this study was obtained from the patient.

### Consent for publication

Informed consent for publication of this case report was obtained from the patient.

### Competing interests

The authors declare that there are no competing interests to report.

## References

[1] Yoshida A Yoshida H Yoshida M Myoepithelioma-like tumors of the vulvar region: a distinctive group of SMARCB1-deficient neoplasms. Am J Surg Pathol 2015; 39: 1102–13. 26171919 10.1097/PAS.0000000000000466

[2] Basnayake O Jayarajah U Seneviratne SA. Endometriosis of the inguinal canal mimicking a hydrocele of the canal of Nuck. Case Rep Surg 2020; 2020: 8849317. 32963875 10.1155/2020/8849317 PMC7495156

[3] Motooka Y Motohara T Honda R Radical resection of an endometrioid carcinoma arising from endometriosis in the round ligament within the right canal of Nuck: a case report and literature review. Gynecol Oncol Rep 2018; 24: 61–4. 29682601 10.1016/j.gore.2018.01.010 PMC5909028

[4] Kaku Y Goto K Kabashima K. Myoepithelioma-like tumor of the vulvar region presenting as a nonmyxoid spindle-cell neoplasm: a potential histologic mimicker of solitary fibrous tumor. Am J Dermatopathol 2016; 38: e87–9. 26959696 10.1097/DAD.0000000000000523

[5] Kojima Y Tanabe M Kato I Myoepithelioma-like tumor of the vulvar region showing infiltrative growth and harboring only a few estrogen receptor-positive cells: a case report. Pathol Int 2019; 69: 172–6. 30737997 10.1111/pin.12765

[6] Xu Y Gao H Gao JL. Myoepithelioma-like tumor of the vulvar region: a case report in China and review of the literature. Diagn Pathol 2020; 15: 3. 31915021 10.1186/s13000-019-0923-0 PMC6950797

[7] Gowripriya G Sridhar K Vij M. Intracranial myoepithelioma: a case report and review of literature. Neurol India 2019; 67: 1347–51. 31744974 10.4103/0028-3886.271273

[8] Hornick JL Fletcher CD. Myoepithelial tumors of soft tissue: a clinicopathologic and immunohistochemical study of 101 cases with evaluation of prognostic parameters. Am J Surg Pathol 2003; 27: 1183–96. 12960802 10.1097/00000478-200309000-00001

[9] Miettinen M McCue PA Sarlomo-Rikala M Sox10—a marker for not only schwannian and melanocytic neoplasms but also myoepithelial cell tumors of soft tissue: a systematic analysis of 5134 tumors. Am J Surg Pathol 2015; 39: 826–35. 25724000 10.1097/PAS.0000000000000398 PMC4431945

[10] Liou SS Memarzadeh S Dry SM A rare case of vulvar extraskeletal myxoid chondrosarcoma: mimics and diagnostic clues. Autops Case Rep 2021; 11: e2021322. 34458187 10.4322/acr.2021.322 PMC8387072

[11] Aminimoghaddam S Sarmadi S Kashian M Unexpectedly large myoepithelioma-like tumor of the vulvar region with two local recurrences: a rare case report and review of the literature. Gynecol Oncol Rep 2023; 50: 101300. 38093797 10.1016/j.gore.2023.101300 PMC10716484

[12] Funovics PT Vaselic S Panotopoulos J The impact of re-excision of inadequately resected soft tissue sarcomas on surgical therapy, results, and prognosis: a single institution experience with 682 patients. J Surg Oncol 2010; 102: 626–33. 20886550 10.1002/jso.21639

